# A new way of monitoring mechanical ventilation by measurement of particle flow from the airways using Pexa method in vivo and during ex vivo lung perfusion in DCD lung transplantation

**DOI:** 10.1186/s40635-018-0188-z

**Published:** 2018-07-27

**Authors:** Ellen Broberg, Martiné Wlosinska, Lars Algotsson, Anna-Carin Olin, Darcy Wagner, Leif Pierre, Sandra Lindstedt

**Affiliations:** 1 Department of Cardiothoracic Anaesthesia and Intensive Care, Skåne University Hospital, Lund University, Lund, Sweden; 2 Department of Cardiothoracic Surgery, Skåne University Hospital, Lund University, Lund, Sweden; 3 Occupational and Environmental Medicine, Department of Public Health and Community Medicine, Institute of Medicine, Sahlgrenska Academy, University of Gothenburg, Gothenburg, Sweden; 4 Experimental Medical Sciences, Lung Bioengineering and Regeneration, Lund University, Lund, Sweden; 5 Wallenberg Center for Molecular Medicine, Lund University, Lund, Sweden

## Abstract

**Background:**

Different mechanical ventilation settings are known to affect lung preservation for lung transplantation. Measurement of particle flow in exhaled air may allow online assessment of the impact of ventilation before changes in the tissue can be observed. We hypothesized that by analyzing the particle flow, we could understand the impact of different ventilation parameters.

**Methods:**

Particle flow was monitored in vivo, post mortem, and in ex vivo lung perfusion (EVLP) in six porcines with the Pexa (particles in exhaled air) instrument. Volume-controlled ventilation (VCV) and pressure-controlled ventilation (PCV) were used to compare small versus large tidal volumes. The surfactant lipids dipalmitoylphosphatidylcholine (DPPC) and phosphatidylcholine (PC) were quantified by mass spectrometry.

**Results:**

In vivo the particle mass in VCV^1^ was significantly lower than in VCV^2^ (*p* = 0.0186), and the particle mass was significantly higher in PCV^1^ than in VCV^1^ (*p* = 0.0322). In EVLP, the particle mass in VCV^1^ was significantly higher than in PCV^1^ (*p* = 0.0371), and the particle mass was significantly higher in PCV^2^ than in PCV^1^ (*p* = 0.0127). DPPC was significantly higher in EVLP than in vivo.

**Conclusions:**

Here, we introduce a new method for measuring particle flow during mechanical ventilation and confirm that these particles can be collected and analyzed. VCV resulted in a lower particle flow in vivo but not in EVLP. In all settings, large tidal volumes resulted in increased particle flow. We found that DPPC was significantly increased comparing in vivo with EVLP. This technology may be useful for developing strategies to preserve the lung and has a high potential to detect biomarkers.

## Background

The use of donation after circulatory death (DCD) donors for lung transplantation (LTX) has received growing interest over the past few years and has recently entered clinical practice [1–3]. DCD donor lungs need to be evaluated prior to transplantation, and the ex vivo lung perfusion (EVLP) method serves as an excellent tool. [4–10]. The donation process of DCD consists of different phases. There is an initial phase when the donor is intubated and mechanically ventilated in the intensive care unit (ICU) unit, a phase when the donor is mechanically ventilated post mortem to optimize the lungs during the waiting time for organ harvesting, a phase when the lungs are evaluated with EVLP to assess whether the graft meets the clinical criteria for donation, and a final phase when the graft is transplanted into the recipient.

The optimal mechanical ventilation setting in the different phases is an area of intense debate. The main method for monitoring airways is by pressure, volume, and airflow. During normal breathing motions, the lung is known to generate particles in the range of 1 μm or less as well as particles up to around 5–10 μm which are exhaled in the breath. These particles are predominantly comprised of surfactants and phospholipids and are thought to originate from the respiratory lining fluid following the opening and closing of the small airways. Monitoring the status of the small airways by analyzing different particle flows online during mechanical ventilation has never been done before, but may provide real-time insight into the effect of changes in mechanical ventilation parameters before changes in conventional parameters can be detected.

In the present study, we used an optical particle counter (OPC) to analyze exhaled particles, particle flow, and their size distribution using the Pexa (particles in exhaled air) method during mechanical ventilation. Pexa has been previously used on non-intubated, awake, normal breathing patients for monitoring airways but has not previously been used during mechanical ventilation. The Pexa method permits real-time monitoring of particle flow as well as collection and subsequent chemical analysis from particles of exhaled air for example phospholipids in asthma patients. Several phospholipids have been identified by time-of-flight secondary ion mass spectrometric analysis of the collected spots in former studies, suggesting that the particles originated from the lower airways; the pattern of phospholipids previously observed are in agreement with that of broncho-alveolar lavage (BAL) [11–15].

We hypothesized that this technology could be useful for monitoring mechanically ventilated patients with respiratory disease and that it may have a high potential to detect biomarkers in exhaled air.

In the present study, the Pexa instrument was customized to be able to use in conjunction with mechanical ventilation. We analyzed the particle flow from porcine airways in vivo, post mortem, and during EVLP using different ventilation modes: volume-controlled ventilation (VCV) and pressure-controlled ventilation (PCV), comparing small tidal volumes versus large tidal volumes. We also analyzed the particle flow during different pulmonary flows and during exposures to vascular drugs such as potassium, norepinephrine, and niprid during EVLP.

## Methods

### Animal preparation

Six Swedish landrace pigs with a mean weight of 61 ± 1.8 kg were fasted overnight with free access to water. Premedication was performed with an intramuscular injection of Xylazine (Rompun® vet. 20 mg/ml; Bayer AG, Leverkusen, Germany; 2 mg/kg) mixed with ketamine (Ketaminol® vet. 100 mg/ml; Farmaceutici Gellini S.p.A., Aprilia, Italy; 20 mg/kg) in their stables, and a peripheral iv access was established in the earlobe. The pig was then transferred to the laboratory and placed in supine position on the operating table. Oral intubation was performed using a 7.5 size endotracheal tube after anesthesia induction with sodium thiopental (Pentothal; Abbott Laboratories, North Chicago, IL, USA) and pancuronium bromide (Pavulon; N.V. Organon, Oss, the Netherlands). Anesthesia was maintained with a ketamine (Ketaminol® vet), midazolam (Midazolam Panpharma®, Oslo, Norway), and fentanyl (Leptanal®, Lilly, France) infusion. Fluid loss was compensated by continuous infusion of Ringer’s Acetate. Mechanical ventilation was established with a Siemens-Elema ventilator (Servo Ventilator 300, Siemens, Solna, Sweden).

### Mechanical ventilation and Pexa measurements

**Fig. 1:**
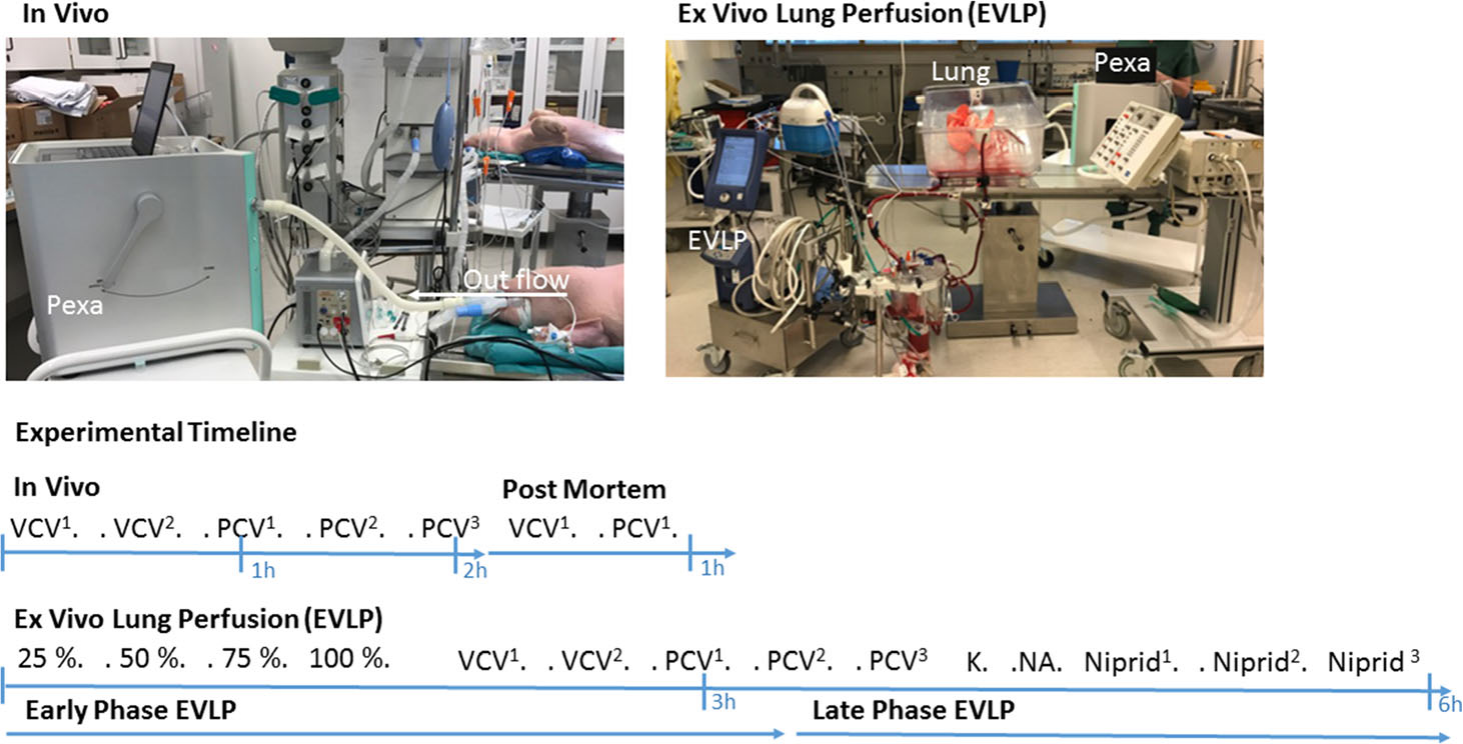
The experimental timeline and the experimental set up in vivo and during ex vivo lung perfusion (EVLP). In the present study, the Pexa instrument has been customized to be able to be used in conjunction with mechanical ventilation. The instrument was connected to the expiratory flow circuit from the animal

The Pexa (particles in exhaled air) 2.0 instrument (PExA, Gothenburg, Sweden) conducts measurements by OPC and has been described previously in patients breathing room air [16]. In the present study, the instrument was customized to be able to be used in conjunction with mechanical ventilation. The instrument samples exhaled particles by impaction and characterized the particle number, concentrations, and size of particles using an OPC. Impactors use the principle of particle inertia to sample particles according to size. The jet of air is directed towards an impaction plate. The impaction plate deflects the flow to give a 90° bend in the streamlines. Particles with inertia above a certain threshold will impact whereas particles below the threshold will follow the streamlines around the impaction plate. For particles of the same density, the particle inertia is mainly determined by particle mass, i.e., size and particle velocity. The size range of particles that impact on the impaction plate can be controlled by adjusting the velocity of the air stream. Since the size ranges of particles that is sampled on the impaction plate is well-defined, the sampled mass of particles on the impaction plate can be calculated from particle measurements made with an OPC. In the present study, the instrument was connected to the outflow air of the mechanical respiratory circuit (Fig. 1). Particles in the diameter size interval of 0.41–4.55 μm, were measured and sampled by the Pexa instrument and referred to as Pex (particles exhaled). Particles in exhaled air were collected using a two stage inertial impactor with 50% cut off diameters of 7.0 μm for the first stage and 0.5 μm for the second stage. Particles between 0.5 and 7.0 μm were sampled onto a thin membrane of hydrophilic polytetrafluoroethylene (FHLC02500, Millipore, Billerica, MA, USA) placed on the second impaction stage. The particles are divided into 8 different size groups with a mean diameter of particle 1; 0.48 μm, particle 2; 0.59 μm, particle 3; 0.75 μm, particle 4; 0.98 μm, particle 5; 1.22 μm, particle 6; 1.67 μm, particle 7; 2.52 μm, particle 8; 3.37 μm. The total accumulated mass (ng) and the total accumulated number of particles (count) from the airways were continuously measured by Pexa instrument during different ventilation modes in vivo. The different ventilation modes all had an inspiratory:expiratory (I:E) ratio of 1:2 on all subjects and were as follows:

1. Volume-controlled ventilation (VCV) with small tidal volumes 6–8 ml/kg, breathing frequency at 16, and PEEP at 2 (VCV^1^).
2. VCV with large tidal volumes 10–12 ml/kg, breathing frequency at 16, and PEEP at 2 (VCV^2^).
3. Pressure-controlled ventilation (PCV) with small tidal volumes 6–8 ml/kg, breathing frequency at 16, and PEEP at 2 (PCV^1^).
4. PCV with large tidal volumes 10–12 ml/kg, breathing frequency at 16, and PEEP at 2 (PCV^2^).
5. PCV with large tidal volumes 10–12 ml/kg, breathing frequency at 16, and PEEP at 10 (PCV^3^).

Each ventilation mode was analyzed during a period of 15 min with periods of restoration in between the settings.

### Experimental timeline

The experimental timeline is demonstrated in Fig. 1.

### Preservation of DCD lungs

A median sternotomy was performed and ventricular fibrillation was induced electrically. The tracheal tube was disconnected from the ventilator when circulatory arrest was confirmed and left open to air. The sternotomy and the skin were temporary closed again and the animals were left untouched for 1 h at room temperature.

#### Ventilation post mortem and Pexa measurements

1 h after the declaration of death, ventilation was re-established. The total accumulated mass (ng) and the total accumulated number of particles (count) from the airways were continuously measured by the Pexa instrument. The number of particles was measured during different ventilation modes in the postmortem animal. The different ventilation modes were as follows: VCV^1^ and PCV^1^ (Fig. 1). Each ventilation mode was analyzed during a period of 15 min with a time period of restoration in between every setting.

One and a half hours after the declaration of death, the median sternotomy was reopened. The pulmonary artery was cannulated via the right ventricle with a 28 F cannula and secured with a purse string suture placed in the outflow tract of the pulmonary artery. The left atrium and inferior vena cava was opened. The lungs were perfused antegradely with Perfadex (XVIVO Perfusion AB, Gothenburg, Sweden).

The cannula was removed from the pulmonary artery. The lungs were harvested en bloc in a standard fashion. After harvesting, the lungs were put on a scale and the lung weight was noted. During the retrieval, a segment (~ 8 cm) of the descending aorta was also excised. The lungs were immersed in cold Perfadex with the aortic segment and put in cold storage at 8° for 1 h.

### Ex vivo lung perfusion

Ex vivo lung perfusion (EVLP) was performed using the extracorporeal perfusion circuit by Medtronics (Medtronic AB, Kerkrade, the Netherlands; Ex Vivo Lung Evaluation Set).

The system was primed with STEEN Solution™ (XVIVO Perfusion AB, Gothenburg, Sweden), and one unit of autologous blood, withdrawn earlier from each donor. Steen solution, Imipenem (0.5 g; Tienam, Merck Sharp & Dohme, Sollentuna, Sweden), insulin (20 IU; Actrapid; Novo Nordisk, Bagsvaerd, Denmark), and heparin (10,000 IU; Leo Pharma, Malmö, Sweden) were added, and isotonic trometamol (Addex-Tham, Kabi, Sweden) was used to buffer the mixed solution to a temperature adjusted to pH of 7.4. Gas was supplied to the membrane oxygenator; first oxygen and CO_2_ during the reconditioning phase, and then 93% nitrogen and 7% CO_2_ during the testing phase, creating a normal venous blood gas in the perfusate to the pulmonary artery (i.e., the oxygenator is used to deoxygenate the perfusate). Before the perfusion was started, the pulmonary artery was prolonged by a segment of the descending aorta to make cannulation easier. The pulmonary artery cannula was then connected to the corresponding tube of the extracorporeal circuit, the air was removed, and the shunt of the circuit was clamped. An endotracheal tube was secured in the trachea with a cotton band and connected to the ventilator. The remnant of the left atrium was left open, prohibiting pulmonary outflow obstruction, and maintaining a constant left atrium pressure around 0 mmHg.

A low-flow perfusion at 25 °C was initiated through the lungs and the lungs were gradually warmed by increasing the temperature of the perfusate. When the temperature reached 32 °C, ventilation was started with a FiO_2_ of 0.5 and a minute volume of 1 l/min, and no positive end-expiratory pressure (PEEP). The pump flow was gradually increased, never allowing the pulmonary arterial pressure to exceed 20 mmHg. After 20–30 min, normothermia was reached and blood gases were analyzed throughout the perfusion. Pulmonary vascular resistance (PVR) was calculated at various points of ventilation using the formula PVR (dyne*s/cm^5^) = (80 * (mean pulmonary artery pressure (MPAP)−(pulmonary capillary wedge pressure (PCWP)))/cardiac output (CO). PCWP, e.g., left atrial pressure (LAP) and CO, e.g., pulmonary artery flow (PAF).

### Mechanical ventilation and Pexa measurements during EVLP

#### Analyses during different pulmonary flow rates

When the lungs reached normothermia and a mean flow rate of 4.0 ± 0.2 was reached, the pulmonary flow was lowered to 25% of the maximum flow and the ventilation was set on VCV with small tidal volumes 6–8 ml/kg, breathing frequency at 16, and PEEP at 2 (VCV^1^). The total accumulated mass (ng) and the total accumulated number of particles (count) from the airways were continuously measured by Pexa instrument. The number of particles was measured during different pulmonary flows during EVLP on 25, 50, 75, and 100% of pulmonary flow respectively. Each pulmonary flow was analyzed during a period of 15 min.

#### Analyses during different ventilation modes

The total accumulated mass (ng) and the total accumulated number of particles (count) from the airways were continuously measured by the Pexa instrument. The number of particles was measured during different ventilation modes ex vivo. The different ventilation modes were as follows: VCV^1^, VCV^2^, PVC^1^, PVC^2^, PVC^3^ respectively, described above. Each ventilation mode was analyzed during a period of 15 min.

#### Analyses during exposure to different drugs

The total accumulated mass (ng) and the total accumulated number of particles (count) from the airways were continuously measured by the Pexa instrument. The ventilation mode was set on VCV^1^. Different drugs were inserted into the EVLP circuit and by the pulmonary circulation exposed to the lungs. Pexa measurements were performed during and after the exposure to potassium 20 mmol (K) norepinephrine 100 μg (NA) and niprid in three different dosage 50, 100, and 150 μg.

### Pulmonary gas function

Blood gases were analyzed in between the different ventilation modes in vivo and ex vivo during the entire experiments.

### Sampling and chemical analysis

The method used for calculating exhaled and sampled particle mass from the measured particle number concentrations has been described previously [14]. Chemical analysis was performed with a high-throughput technique using a triple quadrupole instrument with electrospray ionization operating in positive mode. For quantification of DPPC and PC (16:0 18:2), selected reaction monitoring (SRM) was used.

Samples were extracted using 160 μL of a solvent consisting of methanol, chloroform, and 40 mM ammonium acetate (6:3:2, *v/v/v*) after addition of internal standard (IS). From the extracted sample, 20 μL was injected using a flow gradient injection method with an isocratic mobile phase of methanol, chloroform, and 40 mM ammonium acetate (6:3:2, *v/v/v*). Standard samples were prepared in cryotubes containing Pex sampling membranes spiked with IS before adding a known amount of DPPC and PC (Avanti lipids Alabaster, AL, USA). From standards, a linear regression model using 1/*x* weighing where *y* = analyte_area · IS_area − 1 and *x* = analyte_concentration · IS_concentration − 1 were constructed and used for calculating amounts in unknown samples. Measured DPPC and PC concentrations in exhaled particles were expressed as weight percent, wt%.

### Calculations and statistics

Descriptive statistics, in the form of the number of experimental animals, mean, and the standard error on the mean (SEM) for the different parameters, were analyzed. DPPC and PC results are shown as mean and standard deviation (SD). The results are presented for the different parameters divided into the different groups. Statistically significant difference between the groups was tested with repeated measurement ANOVA. All statistical analyses were performed, using GraphPad Prism. Significance was defined as *p* < 0.001 (***), *p* < 0.01 (**), *p* < 0.05 (*), and *p* > 0.05 (not significant, n.s.).

## Results

### Study groups

Pre-operative partial pressure of oxygen in arterial blood (PaO_2_) at a FiO_2_ of 0.5 was 30.9 ± 0.7 kPa.

No anatomical anomalies, signs of infection, or malignancy were found in any of the animals at autopsy.

### Ventilation in vivo

#### Accumulated particles (ng)—total accumulates mass

**Fig. 2:**
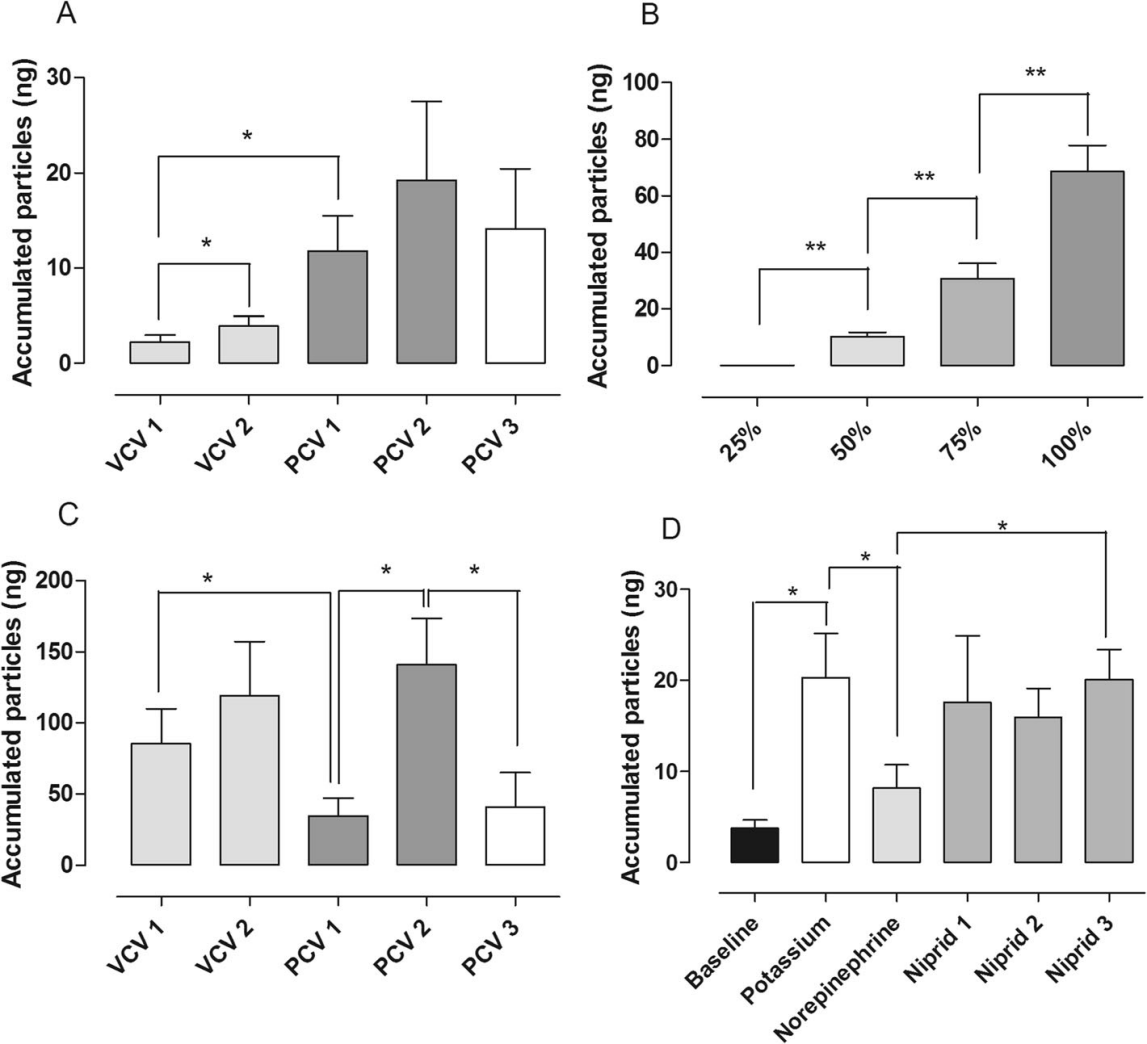
Total accumulated particle mass (ng) measured by Pexa. **a** In vivo, **b** during different pulmonary flow, i.e., percent of cardiac output in ex vivo lung perfusion (EVLP), **c** during different ventilation settings in EVLP, **d** during exposure to different drugs injected into the EVLP circuit. Volume-controlled ventilation (VCV) with small tidal volumes 6–8 ml/kg, breathing frequency at 16, and PEEP at 2 (VCV^1^), VCV with large tidal volumes 10–12 ml/kg, breathing frequency at 16, and PEEP at 2 (VCV^2^), pressure-controlled ventilation (PCV) with small tidal volumes 6–8 ml/kg, breathing frequency at 16, and PEEP at 2 (PCV^1^), PCV with large tidal volumes 10–12 ml/kg, breathing frequency at 16, and PEEP at 2 (PCV^2^), PCV with large tidal volumes 10–12 ml/kg, breathing frequency at 16, and PEEP at 10 (PCV^3^)

The accumulated particle mass from the airways was continuously measured by the Pexa instrument during different ventilation modes. The accumulated particle masses were as follows: VCV^1^ 2.23 ± 0.79 ng, VCV^2^ 3.92 ± 1.04 ng, PCV^1^ 11.75 ± 3.76, PCV^2^ 19.23 ± 8.25, and during PCV^3^ 15.32 ± 7.62. Comparing the different groups, the accumulated particle mass in VCV^1^ was significantly lower than VCV^2^ (*p* = 0.0186), and the accumulated particle mass was significantly higher in PCV^1^ than in the VCV^1^ (*p* = 0.0322). All other comparisons between the groups were found to not be statistically significant. The results are shown in Fig. 2a.

#### Accumulated particles (count)

The accumulated particle mass from the airways was then divided into 8 different groups according to particle size where particle size 1 is the smallest and particle size 8 is the largest particle. The results are shown in Table 1.

**Table 1:** Shows total particle count for the different particle sizes from 1 to 8 during in vivo ventilation.

	In vivo							
	VCV versus PVC			Small tidal volumes versus large tidal volumes		Low PEEP versus high PEEP		
	VCV^1^	PCV^1^	*p* value	VCV^2^	*p* value	PCV^2^	PCV^3^	*p* value
Particle 1	1650 ± 277	11,543 ± 3044	0.02*	6300 ± 1197	0.003**	18,428 ± 3044	15,387 ± 2350	n.s.
Particle 2	992 ± 208	7521 ± 2099	0.03*	4238 ± 793	0.002**	12,580 ± 1288	10,743 ± 1983	n.s.
Particle 3	1683 ± 286	10,455 ± 2481	0.06	4948 ± 918	0.009**	15,125 ± 2069	15,245 ± 1937	n.s.
Particle 4	1233 ± 159	7208 ± 651	0.01*	4130 ± 955	0.019*	6066 ± 1299	10,003 ± 2331	n.s.
Particle 5	138 ± 28	3025 ± 437	0.02*	1345 ± 390	0.029*	3890 ± 1944	3025 ± 1151	n.s.
Particle 6	350 ± 32	3766 ± 476	0.01*	1552 ± 370	0.028*	4245 ± 1461	3570 ± 1029	n.s.
Particle 7	175 ± 56	2018 ± 259	0.01*	1255 ± 451	0.046*	2137 ± 762	1723 ± 735	0.008*
Particle 8	92 ± 20	2293 ± 376	0.02*	1053 ± 485	n.s.	2133 ± 1059	1426 ± 687	n.s.

*VCV1* volume-controlled ventilation with small tidal volumes and PEEP at 2, *VCV2* volume-controlled ventilation with large tidal volumes and PEEP at 2, *PCV1* pressure-controlled ventilation with small tidal volumes and PEEP at 2, *PCV2* pressure-controlled ventilation with large tidal volumes and PEEP at 2, *PCV3* pressure-controlled ventilation with big tidal volumes and PEEP at 10

Significance was defined as: *p* < 0.01 (**), *p* < 0.05 (*), and *p* > 0.05 (not significant, n.s.)

### Ventilation post mortem

#### Accumulated particles (ng)—total accumulates mass

The accumulated particle mass from the airways was again continuously measured by the Pexa instrument during different ventilation modes postmortem. The number of particles was as follows: VCV^1^ 0.50 ± 0.22 ng, and at VCV^2^ 0.67 ± 0.21 ng (*p* = n.s.).

### Ex vivo lung perfusion

#### During different pulmonary flow

##### Accumulated particles (ng)—total accumulates mass

The number of particles from the airways of ex vivo lungs was continuously measured by the Pexa instrument. The total accumulated mass was measured during different pulmonary flows. The ventilation was kept at volume controlled ventilation with a small tidal volumes 6–8 l/kg, breathing frequency at 16, and PEEP at 2 (VCV^1^). The total accumulated mass was as follows: 25% of the total pulmonary flow was 0 ± 0 ng, 50% 10.33 ± 1.53, 75% 30.67 ± 5.36, and at 100% 68.67 ± 9.24. Comparing the different groups, the total accumulated mass at 50% pulmonary flow was significantly higher than at 25% pulmonary flow (*p* = 0.0013), and the total accumulated mass at 75% pulmonary flow was significantly higher than at 50% pulmonary flow (*p* = 0.0089), furthermore the total accumulated mass at 100% pulmonary flow was significantly higher than at 75% pulmonary flow (*p* = 0.0039) as seen in Fig. 2b.

### EVLP at different ventilation mode

#### Accumulated particles (ng)—total accumulates mass

The accumulated particle mass from the airways of ex vivo lungs was continuously measured by the Pexa instrument. The accumulated particle mass was assessed during different ventilation modes. The number of particles was as follows: VCV^1^ 85.53 ± 24.22 ng, VCV^2^ 119.17 ± 38.03 ng, PCV^1^ 34.67 ± 12.37 ng, PCV^2^ 140.87 ± 32.54 ng, and at PCV^3^ 17.00 ± 5.83 ng. Comparing the different groups, the accumulated particle mass in VCV^1^ was significantly higher than PCV^1^ (*p* = 0.0371), the accumulated particle mass was significantly higher in PCV^2^ than in the PCV^1^ (*p* = 0.0127) and the accumulated particle mass was significantly higher in PCV^2^ than in the PCV^3^ (*p* = 0.0499). All other comparisons between the groups were found not to be statistically significant. The results are shown in Fig. 2c.

#### Accumulated particles (count)

The accumulated particle mass from the airways was then divided into 8 different groups according to particle size where particle 1 is the smallest and particle 8 is the biggest particle. The results are shown in Table 2.

**Table 2:** Shows total particle count for the different particle sizes from 1 to 8 during ex vivo ventilation.

	Ex vivo lung perfusion							
	VCV versus PVC			Small tidal volumes versus large tidal volumes		Low PEEP versus high PEEP		
	VCV^1^	PCV^1^	*p* value	VCV^2^	*p* value	PCV^2^	PCV^3^	*p* value
Particle 1	141,087 ± 36,170	51,135 ± 31,907	0.020*	224,313 ± 94,259	0.018*	177,117 ± 41,998	23,258 ± 5060	0.020*
Particle 2	150,750 ± 208	57,818 ± 33,823	0.022*	239,288 ± 95,435	0.026*	222,225 ± 56,596	23,913 ± 5575	0.025*
Particle 3	192,577 ± 46,145	76,725 ± 41,409	0.014*	316,550 ± 120,963	0.028*	308,437 ± 80,941	34,092 ± 8124	0.027*
Particle 4	106,700 ± 35,066	43,818 ± 21,247	0.021*	168,932 ± 63,215	0.028*	186,240 ± 49,551	19,900 ± 4730	0.029*
Particle 5	35,373 ± 12,136	14,056 ± 6778	0.020*	54,517 ± 21,202	0.034*	60,478 ± 19,729	8052 ± 2514	0.032*
Particle 6	23,542 ± 8709	7657 ± 3532	0.034*	33,600 ± 12,443	0.048*	35,550 ± 12,188	5486 ± 1543	0.038*
Particle 7	7070 ± 2883	2388 ± 1097	n.s.	10,107 ± 3723	n.s.	8497 ± 2997	2018 ± 538	0.536
Particle 8	3340 ± 1282	722 ± 222	n.s.	3757 ± 1354	n.s.	3000 ± 977	790 ± 262	0.045*

*VCV1* volume-controlled ventilation with small tidal volumes and PEEP at 2, *VCV2* volume-controlled ventilation with large tidal volumes and PEEP at 2, *PCV1* pressure-controlled ventilation with small tidal volumes and PEEP at 2, *PCV2* pressure controlled ventilation with large tidal volumes and PEEP at 2, *PCV3* pressure-controlled ventilation with big tidal volumes and PEEP at 10

Significance was defined as: *p* < 0.05 (*), and *p* > 0.05 (not significant, n.s.)

### EVLP—influence of different drugs

#### Accumulated particles (ng)—total accumulates mass

The accumulated particle mass from the airways was continuously measured by the Pexa instrument during administration of different medications. The accumulated particle mass was measured during different ventilation modes. The amount of particles was as follows: baseline 3.80 ± 0.90 ng, potassium (K) 20.33 ± 4.85 ng, after norepinephrine (NA) 8.17 ± 2.60 ng, after niprid 1 (50 μg) 17.61 ± 7.31 ng, after niprid 2 (100 μg) 15.96 ± 3.16 ng, and after niprid 3 (150 μg) 20.08 ± 3.33 ng. The accumulated particle mass was significantly higher following administration of potassium as compared to baseline (*p* = 0.0268), the accumulated particle mass was significantly lower after administration of norepinephrine than after administration of potassium (*p* = 0, 0285) and the accumulated particle mass was significantly higher after administration of 150 μg niprid than after administration of norepinephrine (*p* = 0.0349). All other comparisons between the groups were found not to be statistically significant. The results are shown in Fig. 2d.

#### Accumulated particles (count)

The accumulated particle mass from the airways was then divided into 8 different groups according to particle size where particle 1 is the smallest and particle 8 is the largest particle. The results are shown in Table 3.

**Table 3:** Shows total particle count for the different particle sizes from 1 to 8 during ex vivo lung perfusion ventilation during exposure to different drugs

	Exposure to different drugs during ex vivo lung perfusion						
	Baseline	Potassium	*p* value	Norephineprine	*p* value	Niprid^3^	*p* value
Particle 1	3116 ± 501	34,651 ± 14,996	0.031*	26,477 ± 10,248	n.s.	51,112 ± 33,163	n.s.
Particle 2	3402 ± 699	43,478 ± 18,622	0.028*	30,545 ± 13,340	n.s.	70,152 ± 49,852	n.s.
Particle 3	5367 ± 1033	60,670 ± 24,601	0.047*	42,213 ± 16,477	n.s.	107,173 ± 74,085	n.s.
Particle 4	4649 ± 1140	37,785 ± 15,403	0.018*	25,788 ± 10,395	n.s.	71,795 ± 47,225	n.s.
Particle 5	1541 ± 502	13,298 ± 5487	n.s.	8900 ± 3438	n.s.	27,202 ± 17,516	n.s.
Particle 6	1112 ± 354	8500 ± 2967	n.s.	5643 ± 1982	n.s.	17,453 ± 11,027	n.s.
Particle 7	238 ± 87	2655 ± 895	0.048*	1757 ± 584	n.s.	5118 ± 3260	n.s.
Particle 8	221 ± 68	1182 ± 437	n.s.	908 ± 368	n.s.	2373 ± 1539	n.s.

Significance was defined as: *p* < 0.05 (*), and *p* > 0.05 (not significant, n.s.)

### Pulmonary gas function

The pulmonary gas function and blood gases were analyzed between every different modes: in vivo; baseline, VCV^1^, VCV^2^, PCV^1^, PCV^2^, PCV^3^, and during ex vivo lung perfusion; baseline, VCV^1^, VCV^2^, PCV^1^, PCV^2^, and PCV^3^. The pulmonary gas function, blood gases, were also analyzed between every different mode in ex vivo lung perfusion between exposure to different drugs; baseline, potassium, norepinephrine, niprid 50 μg, niprid 100 μg, and niprid 150 μg. All lungs had excellent blood gases during the whole experiments. No significant differences were found between the different settings.

### Hemodynamic data during EVLP

#### Pulmonary artery flow (PAF) and mean pulmonary artery pressure (MPAP)

PAF, i.e., CO in the ex vivo model and MPAP was measured continuously. The PAF was not allowed to exceed 4.0 l/min, and the MPAP was not allowed to exceed 20 mmHg.

#### Pulmonary vascular resistance (PVR)

PVR was calculated using the following formula: PVR (dyne × s/cm5) = (80 * (MPAP − PCWP))/CO, where PCWP is equivalent to LAP and CO is equivalent to PAF in the EVLP method.

Hemodynamics and blood gases during in vivo and ex vivo are shown in Table 4.

**Table 4:** Shows the hemodynamics, and blood gases during in vivo early phase and late phase, and during ex vivo lung perfusion (EVLP) in the start and of early phase and late phase

	In vivo	In vivo	EVLP	EVLP	EVLP	EVLP
	Early phase	Late phase	Early phase start	Early phase end	Late phase start	Late phase end
Heart rate (bmp)	91 ± 21	92 ± 18				
Systolic blood pressure (mmHg)	89 ± 18	88 ± 19				
Diastolic blood pressure (mmHg)	65 ± 16	67 ± 15				
Temperature (°C)	36.8 ± 0.9	36.8 ± 0.9	37 ± 0.1	37 ± 0.1	37 ± 0.1	37 ± 0.1
SpO_2_ (%)	99 ± 1.0	99 ± 1.0	99 ± 1	99 ± 1	99 ± 1	99 ± 1
pH	7.5 ± 0.2	7.5 ± 0.2	7.5 ± 0.1	7.5 ± 0.2	7.5 ± 0.1	7.5 ± 0.2
P_o2_ (mmHg)	30.9 ± 0.7	31.2 ± 0.9	64.8 ± 6.0	65.6 ± 5.0	67.7 ± 1.8	63.3 ± 2.3
P_co2_ (mmHg)	3.87 ± 0.23	3.86 ± 0.24	2.9 ± 0.3	3.0 ± 0.4	3.2 ± 0.6	3.1 ± 0.9
FiO_2_	0.5	0.5	1.0	1.0	1.0	1.0
Flowrate (L/min), i.e., cardiac output (CO)			3.5 ± 0.1	3.5 ± 0.1	3.4 ± 0.5	2.5 ± 0.5
Pulmonary pressure (mmHg)			12 ± 0.5	11 ± 0.4	15 ± 1.5	19 ± 1.0
Left atrium pressure (mmHg)			0 ± 0	0 ± 0	0 ± 0	0 ± 0
Pulmonary vascular resistance (PVR)			273 ± 12	251 ± 2	361 ± 23	623 ± 36

### DPPC and PC concentration in PEx

DPPC and PC concentrations were measured in exhaled particles of four animals and were expressed as weight percent (wt%). The amount DPPC in percent (wt%) of total mass of the Pex sample is shown in Fig. 6a. Note the significant increase in DPPC in EVLP late phase as compared to in vivo (*p* = 0.04). No differences were observed in the PC in wt% of total Pex sample, as shown in Fig. 6b.

## Discussion

Lung-protective ventilation is a commonly used strategy for mechanical ventilation after lung transplantation. An international survey showed that PCV mode was used in 37% of cases and VCV mode in 35% of cases [17]. In our clinical practice, we use predominantly PCV types of modes and therefore we focused the current study on PCV modes. Here, we introduce a new technique to measure particle flow from the airways during mechanical ventilation. Particle flow from the airways could be used as a non-invasive method for evaluating the different possible settings used in mechanical ventilation. The Pexa instrument was customized to be able to be used in conjunction with mechanical ventilation. In the present study, we analyzed different particle flows during different ventilation modes in LTX DCD, i.e., in vivo, post mortem, and ex vivo.

The Pexa technique has not been used in mechanical ventilation before, and we wanted to try to achieve a situation that would most likely demonstrate a difference in particle flow between different ventilation modes and tidal volumes. We assumed that with having a low PEEP as a basic setting, we could facilitate the detection of the largest change between small and large tidal volumes in particle flow. It is, however, generally more preferable to use a higher PEEP (e.g., five PEEP) in clinical practice. In vivo, we found that VCV resulted in a significantly lower particle flow than PCV; we also found that large tidal volumes resulted in a larger particle flow than small tidal volumes. Increasing PEEP from 2 to 10 did not result in any significant increase or decrease in particle flow in the in vivo ventilation; however, after we went from 10 PEEP to 2 PEEP, we observed an increase in particle flow from the airways. The overall observation was that the particle flow was higher in all PCV settings using small or large tidal volumes, high or low PEEP. The difference in particle flow between the different ventilation modes and between small and large tidal volumes might be suggested to be caused by an increased opening and closing of small lung segments during PCV and during large tidal volumes.

The second phase of the DCD donation is mechanical ventilation post mortem. While there is no circulation present at that time, the lungs are normally ventilated with small tidal volumes at low or no PEEP. In the present study, we compared VCV^1^ with PCV^1^. None of the ventilation modes resulted in a considerable difference in particle flow from the small airways.

**Fig. 3:**
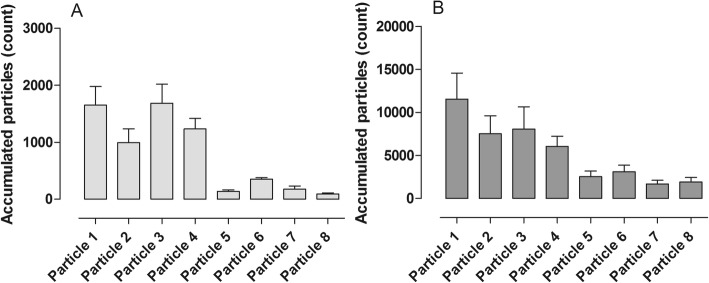
Distribution of different particle sizes during different ventilation modes in vivo. **a** Volume-controlled ventilation (VCV) and **b** pressure-controlled ventilation (PCV). Note the differences in the total amount of particle count in the two different ventilation settings; interestingly, the same particle size distribution was seen in the both settings

In the third phase of our study, the lungs were harvested and after 1 h in cold storage, the lungs were connected to the ex vivo lung perfusion and allowed to warm up gradually. During DCD lung donation and the lungs brought to clinical grade with EVLP, it is generally thought that the EVLP phase is where the donor lung is most susceptible to lung injury due to the use of positive pressure ventilation and the fact that the chest cavity is no longer present to limit distension. Therefore, it is of intense interest to better understand what is occurring during this phase and to better understand how to reduce potential lung injury. In our study, we warmed the lungs and ventilation together until we reached full pulmonary flow of 3.5–4 l/min and full ventilation according to VCV^1^ ventilation mode. The ventilation mode was then kept stable, and the pulmonary flow was put to 25, 50, 75, and 100% subsequently. When only 25% of the pulmonary flow was driven through the lung, we did not see any particular flow from the small respiratory tracts. As pulmonary flow increased, the flow of particles from the small respiratory tracts also significantly increased. This suggests that the particle flow is somehow at least partly dependent on blood flow through the lungs. Figure 3b displays the stepwise increase in particle flow from the airways when blood flow through the lungs is increased stepwise.

The fact that particle flow increases with increased blood flow might also explain why there was an increased particle flow when going from PEEP 10 to PEEP 2. PEEP 10 might have decreased the blood flow in the lungs while with PEEP 2 the blood flow can be suggested to have increased and thereby also increased the particle flow in exhaled air. We could not detect any significant change in increased airway pressure during the EVLP process relating to particle flow changes. There was also no considerable particle flow in any ventilation mode during post mortem ventilation which gives some evidence for the fact that blood flow through the lung is of some importance for particle flow in exhaled air. This indicates that the particle flow in exhaled air might reflect the blood flow through the lung and particularly the small airways. This observation warrants further study. Importantly, we did not observe any correlation between particle collection and results from blood gases in the EVLP phase of our study and nor from the in vivo and post mortem phases either.

Different settings of ventilation modes were then analyzed and again we found that large tidal volume resulted in an increased particle flow from the airways compared to small tidal volumes. However, we did not observe a lower particle flow in VCV as compared to PCV. The relationship was rather the reverse for small tidal volumes, where PCV^1^ resulted in a significantly lower particle flow from the respiratory tract than VCV^1^. No difference was found between the particle flows in the small airways for large tidal volume, i.e., VCV^2^ and PCV^2^. We anticipated that small PEEP would produce a significantly higher particle flow from the airways than high PEEP during in vivo ventilation. However, we noticed that when releasing PEEP from 10 to 2, i.e., high PEEP to low PEEP, this resulted in a significant increase of particle flow from the respiratory tract. We believe that this suggests that blood flow through the lung might play a significant role for particle flow in exhaled air and particle flow may reflect blood flow through the lung. We observed different patterns between in vivo and EVLP but not between different ventilation modes, i.e., PCV and VCV. Differences were only observed between small and large tidal volumes. This may be due to the fact that there is an underlying physiological difference which can be detected with the Pexa technique between the in vivo and EVLP setting since the thoracic wall is not there; no difference was seen between PCV and VCV but only between small and large tidal volumes.

The current experiment was divided into in vivo, EVLP early phase and EVLP late phase. We found that DPPC was significantly increased comparing in vivo with EVLP late phase. The experimental protocol was rather long, and the EVLP late phase reflects EVLP after up to 6 h in conjunction with demanding ventilation with intervals of tidal volumes of 10–12 ml/kg. In the end of late phase of EVLP, the PVR significantly increased indicating decreased pulmonary function (Table 4). Interestingly, in the majority of animals, we saw raised pulmonary artery pressure and PVR. The significant increase in DPPC might be a sign of lung parenchyme injury. Interestingly, comparing early and late phase EVLP, there was a significantly smaller amount of particle counts in the late phase compared to the early phase. Assuming that the particle flow mostly comes from the small respiratory tract and that a lung circulated in the EVLP cannot continuously reproduce the components of lining fluid in the small respiratory tracts, this might reflect a depletion of surfactants in the lung. When the amount of surfactant is depleted, the lung is known to be more susceptible to damage. Possibly, for this reason, it may be beneficial to add surfactant to the EVLP at longer preservation times for the maintenance of lung tissue homeostasis or for ex vivo regeneration.

**Fig. 4:**
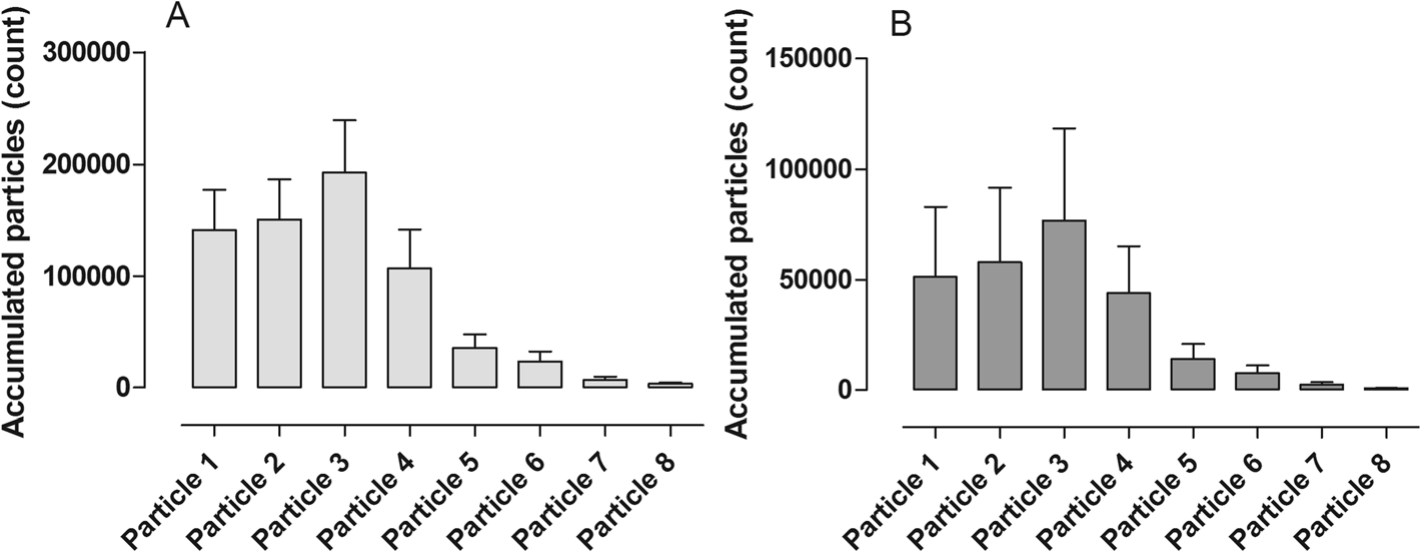
Distribution of different particle sizes during different ventilation modes in ex vivo lung perfusion (EVLP). **a** Volume-controlled ventilation (VCV), and **b** pressure-controlled ventilation (PCV). Note the differences in the total amount of particle count in the two different ventilation settings; interestingly, the same particle size distribution was seen in the both settings

**Fig. 5:**
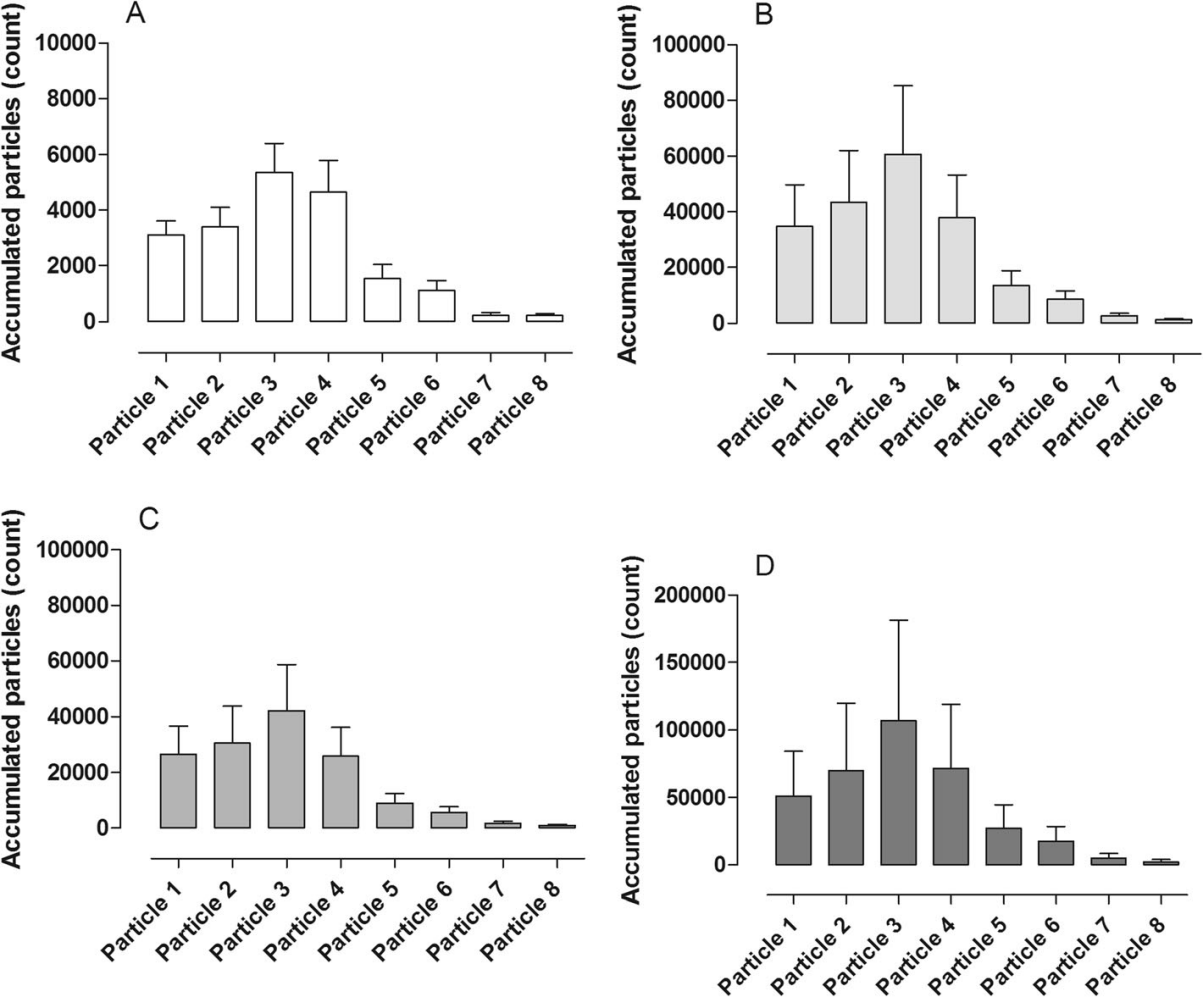
Different drugs were injected into the ex vivo lung perfusion (EVLP) circuit. Distribution of different particle sizes at **a** baseline, and after injection of **b** potassium (K), **c** norepinephrine (NA), and **d** niprid. Note the differences in the total amount of particle count in the different settings; interestingly, the same particle size distribution was seen in all the settings. Volume-controlled ventilation (VCV) with small tidal volumes 6–8 ml/kg, breathing frequency at 16, and PEEP at 2 (VCV^1^) was used during all settings

The Pexa method is a new technology and differs from other technologies used to find biomarkers in exhaled air such as capillary gas chromatography [18]. The Pexa technique uses an optical particle counter for particle flow count and mass spectrometry (MS) for biomarker analysis. This technology gives the opportunity to both measure total mass of all particles but also different particle sizes, divided into eight size groups. See Fig. 2 for example of measurement of total mass of all particles and Figs. 3, 4, and 5 for example of distribution according to different particle sizes. There is only one study that has studied proteins in exhaled air with the Pexa technique in patients which have received a lung transplant, but these patients were breathing room air and were not on mechanical ventilation. The study showed that surfactant A levels in 4 of the 7 patients who developed BOS at 18 months post-transplantation were in the same range as that of healthy controls and of those patients who did not develop bronchiolitis obliterans syndrome [19]. The particles from the airways were collected onto a substrate and analyzed for the phospholipids DPPC and PC, which are major components of lung surfactant. Our study demonstrates the feasibility of measuring phospholipids in exhaled breath particles during mechanical ventilation which could be an important endpoint in evaluating different ventilation schemes.

Concerning the hypothesis that small tidal volumes are likely to be gentler for the lungs, it is very interesting to see that VCV showed a more favorable pattern during in vivo ventilation then PCV. It is unclear why this may be the case, but it may be due to the fact that at this stage of the study, the lungs were perfectly healthy. VCV might just keep the lungs more optimally open in the distal airways since the pigs have not had time to develop the common complications of mechanical ventilation such as atelectasis, volume trauma, or barotrauma.

One interesting finding from our study is that large tidal volumes gave larger particle flow than small tidal volumes in all modes and all settings. This suggests that small particle flow might be associated with better preservation of normal lung tissue function than large particle flow. Large tidal volume has been previously shown to be more traumatic and is associated with worse outcomes; this might support the hypothesis that larger particle flow is worse than small particle flow [20].

In this study, we also attempted to alter the capillary wall permeability with the help of selected drugs to alter the dilation and constriction of the capillary bed by using K for vasodilatation followed by NA for vasoconstriction and niprid for vasodilatation after the NA vasoconstriction. When this was performed, we could see a statistically significant increase in the number of particles going from baseline to vasodilatation and from vasoconstriction to vasodilatation. This might be due to the fact that blood flow and change in capillary wall permeability plays an important role on the particle flow which may be a physiological sign for the importance of pulmonary blood flow for the function and status condition of the lung parenchyma.

**Fig. 6:**
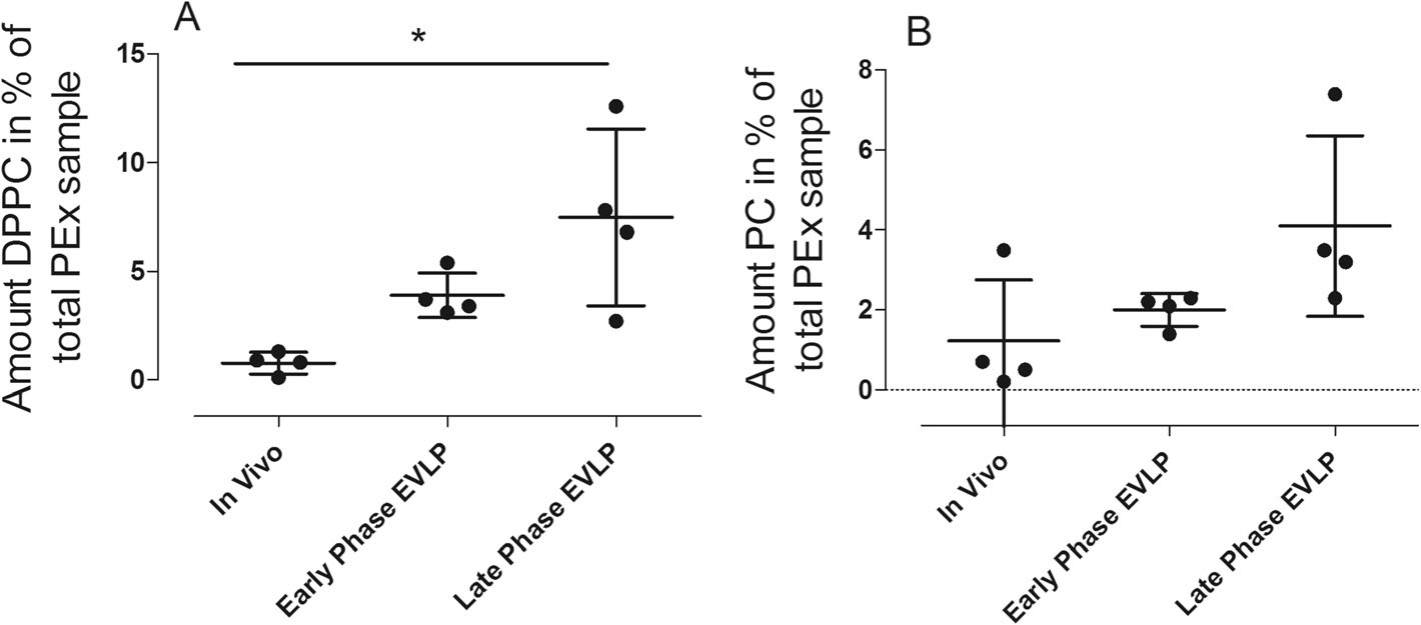
DPPC and PC concentrations were measured in exhaled particles in four of the animals and were expressed as weight percent (wt%). The amount DPPC in percent (wt%) of total Pex sample is shown in **a**. Note the significant increase in DPPC in EVLP late phase as compared to in vivo (*p* = 0.04). No differences were observed in the PC wt% of total Pex sample, as shown in **b**

We have found that the Pexa method can be used to assess the ventilation from the small airways by studying the pattern of particles during different ventilation modes in different ventilation settings. While we used Pexa in the current study for LTX DCD, it can most likely be applicable for other conditions such as infections and ARDS. Using conventional techniques, it is difficult to assess at the bedside if the lung is damaged in any possible way and more so which parts of the lung that are damaged. Previously, the lack of techniques to assess potential early markers of damage made it difficult to evaluate what ventilation modes and settings would be most appropriate in each setting of lung damage. Real-time Pexa analysis may provide new opportunities for monitoring changes in ventilation parameters in individual patients. This technique could be clinically implemented in the future after further studies and could provide a means to individualize the mechanical ventilation parameters for patients with respect to ventilation mode, PEEP settings, and possible evaluation of different tidal volumes. With regard to EVLP procedures, this technique could possibly make use of biomarkers in the sense of discriminating which lungs are suitable for transplantation (Fig. 6).

### Limitations

There are several factors and conditions that limit the impact of this study. This study is of pure experimental character and has been conducted in a small population of healthy animals in a controlled setting. In subjects with ongoing or underlying lung injury, the results may be dramatically different. Therefore, the results and implementations of the findings have to be considered with this in mind. Nonetheless, these initial studies indicate the potential of the Pexa technique for helping to generate further important knowledge both to the physiology of the lung during mechanical ventilation and also changes of the lung during mechanical ventilation. We anticipate that this technique and our results will be applicable to subjects with lung injury, but further studies need to be performed.

We have not collected or attempted to study if any particles have been deposited on the inside lining of the endotracheal tube or other parts of the respiratory circuit. In this study, one could assume a proportionate deposition of particles on the entire respiratory circuit between the different pigs, since all the pigs used the same sized endotracheal tube and type of respiratory circuit. We cannot definitively exclude that the changes we observe in particle amounts or shifts in particle size distributions between the different conditions are only due to changes in the lung. These technical aspects strongly need to be considered in future studies. In this study, we assume that some degree of lung injury has occurred because of the experimental parameters, but we have not formally assessed whether or not this has actually occurred. Future studies which evaluate transpulmonary pressure changes, lung surface tension changes, or histological changes in the tissue will be important to incorporate in future studies to validate changes in particle flow with lung injury status.

To our knowledge, there has only been a few published studies on the Pexa technique and they are all done on patients breathing room air and not on mechanical ventilation. That data however clearly shows that surfactants, lipids, and albumins can be collected by this system, but of course, we can only assume that the same system will be able to detect these particles during a different setting, such as with mechanical ventilation as in our study, but there is no 100% certainty [12, 14, 21, 22].

## Conclusions

Here, we have established a new method for measuring particle flow from the airways during mechanical ventilation. We have also shown that exhaled particles can be collected and analyzed. Generally, VCV resulted in a lower particle flow from the airways in vivo but not in EVLP. In all settings large tidal volumes resulted in an increase of particle flow compared to small tidal volumes. We believe this technology will be useful for monitoring and establishing individual mechanical ventilation parameters which will aid in preserving the lung quality. The technology also has a high potential to detect biomarkers in exhaled air.

## Abbreviations

ARDS: Acute respiratory distress syndrome
BAL: Brocho-alveolar lavage
CO: Cardiac output
DCD: Donation after circulatory death
DPPC: Dipalmitoylphosphatidylcholine
EVLP: Ex vivo lung perfusion
I:E: Inspiratory:expiratory
ICU: Intensive care unit
IS: Internal standard
K: Potassium
LAP: Left atrium pressure
LTX: Lung transplantation
MPAP: Mean pulmonary artery pressure
NA: Norepinephrine
OPC: Optical particle counter
PAF: Pulmonary artery flow
PaO2: Partial pressure of oxygen in arterial blood
PC: Phosphatidylcholine
PCV: Pressure-controlled ventilation
PCWP: Pulmonary capillary wedge pressure
PEEP: Positive end-expiratory pressure
Pex: Particles exhaled
Pexa: Particles in exhaled air
PVR: Pulmonary vascular resistance
SD: Standard deviation
SEM: Standard error on the mean
SRM: Selected reaction monitoring
VCV: Volume-controlled ventilation
wt%: Weight percent
